# Phorbaketal A, Isolated from the Marine Sponge *Phorbas* sp., Exerts Its Anti-Inflammatory Effects via NF-κB Inhibition and Heme Oxygenase-1 Activation in Lipopolysaccharide-Stimulated Macrophages

**DOI:** 10.3390/md13117005

**Published:** 2015-11-19

**Authors:** Yun-Ji Seo, Kyung-Tae Lee, Jung-Rae Rho, Jung-Hye Choi

**Affiliations:** 1 Department of Life & Nanopharmaceutical Sciences, Kyung Hee University, Seoul 130-701, Korea; E-Mails: syg9108@khu.ac.kr (Y.-J.S.); ktlee@khu.ac.kr (K.-T.L.); 2 College of Pharmacy, Kyung Hee University, Seoul 130-701, Korea; 3 Department of Marine Biotechnology, Kunsan National University, Jeonbuk 573-701, Korea; E-Mail: jrrho@kunsan.ac.kr

**Keywords:** phorbaketal A, macrophages, nitric oxide, prostaglandin E_2_, inflammatory cytokine, NF-κB, HO-1

## Abstract

Marine sponges harbor a range of biologically active compounds. Phorbaketal A is a tricyclic sesterterpenoid isolated from the marine sponge *Phorbas* sp.; however, little is known about its biological activities and associated molecular mechanisms. In this study, we examined the anti-inflammatory effects and underlying molecular mechanism of phorbaketal A in lipopolysaccharide (LPS)-stimulated RAW 264.7 macrophages. We found that phorbaketal A significantly inhibited the LPS-induced production of nitric oxide (NO), but not prostaglandin E_2_, in RAW 264.7 cells. Further, phorbaketal A suppressed the expression of inducible NO synthase at both the mRNA and protein levels. In addition, phorbaketal A reduced the LPS-induced production of inflammatory cytokines such as tumor necrosis factor-alpha, interleukin (IL)-1beta, IL-6, and monocyte chemotactic protein-1. Treatment with phorbaketal A inhibited the transcriptional activity of nuclear factor-kappaB (NF-κB), a crucial signaling molecule in inflammation. Moreover, phorbaketal A up-regulated the expression of heme oxygenase-1 (HO-1) in LPS-stimulated RAW 264.7 cells. These data suggest that phorbaketal A, isolated from the marine sponge *Phorbas* sp., inhibits the production of inflammatory mediators via down-regulation of the NF-κB pathway and up-regulation of the HO-1 pathway.

## 1. Introduction

Inflammation is a crucial defense mechanism against pathogens and various external stimuli [1]. Macrophages play an important role in inflammatory and other immune processes. When macrophages are activated by external stimuli, they produce and secrete numerous endogenous inflammatory mediators, including prostaglandin E_2_ (PGE_2_), nitric oxide (NO), and pro-inflammatory cytokines [2]. These pro-inflammatory cytokines, including tumor necrosis factor-alpha (TNF-α), interleukin (IL)-1beta (1β), IL-6, and monocyte chemotactic protein-1 (MCP-1), have pleiotropic effects on immune responses and acute-phase reactions [3]. Therefore, modulation of inflammatory mediators produced by macrophages is an important target for the treatment of inflammation.

Aberrant activity of the transcription factor nuclear factor-kappaB (NF-κB) is related to various inflammatory diseases, including inflammatory bowel disease, arthritis, sepsis, and gastritis [4]. NF-κB has been implicated in the expression of several genes encoding mediators of immune and inflammatory responses. In macrophages, inflammatory stimuli activate the NF-κB pathway, leading to the secretion of pro-inflammatory mediators such as NO, PGE_2_, TNF-α, IL-1β, IL-6, and MCP-1 [5]. In unstimulated cells, subunits p50 and p65 of NF-κB are sequestered in the cytoplasm as complexes with a family of inhibitors known as inhibitors of κB (IκB). NF-κB activation is dependent on the phosphorylation of IκB; this process results in the degradation of IκB and the translocation of NF-κB to the nucleus [6].

Heme oxygenase-1 (HO-1) catalyzes the conversion of heme into carbon monoxide, biliverdin, and Fe^2+^. Many studies have indicated anti-inflammatory and anti-oxidative roles for HO-1 and its enzymatic products [7,8,9]. For example, in activated macrophages, HO-1 and its products have been shown to exert anti-inflammatory effects via attenuation of the expression of pro-inflammatory mediators such as NO, PGE_2_, TNF-α, IL-1β, IL-6, and MCP-1 [10,11,12,13]. Nuclear transcription factor erythroid 2-related factor 2 (Nrf2) regulates the inducible expression of HO-1 at the transcriptional level. In response to oxidative stress, Nrf2 translocates to the nucleus and binds anti-oxidant-related elements in the promoter region of target genes, including HO-1 [14]. Nrf2 has been shown to be an integral part of cytoprotection against inflammation, reactive oxygen species (ROS), and cellular damage [15].

Natural marine products have been reported to have a wide range of therapeutic effects, including anti-inflammatory, antimicrobial, antihypertensive, anticancer, and immune modulatory effects [16,17,18,19,20,21]. Among marine invertebrates, marine sponges are a major source of structurally novel compounds for use in the development of therapeutic products [22,23,24,25]. In particular, the genus *Phorbas* has been reported to produce diverse and potent biologically active components with unique structures [26].

In a recent study, we isolated the tricyclic sesterterpenoid phorbaketal A from *Phorbas* sp., for the first time, and demonstrated its cytotoxicity against human cancer cells [27]. In addition, phorbaketal A was reported to regulate osteoblast and adipocyte differentiation via the protein transcriptional coactivator with PDZ-binding motif (TAZ) [28,29]. Few studies evaluated the biological activities of phorbaketal A, and thus its molecular mechanism remains poorly understood. In particular, the possible anti-inflammatory effects of phorbaketal A have never been demonstrated. Thus, in this study, we investigated the anti-inflammatory activities of phorbaketal A and its molecular mechanism in lipopolysaccharide (LPS)-induced RAW 264.7 macrophages.

## 2. Results and Discussion

### 2.1. Phorbaketal A Inhibits LPS-Induced NO Production and iNOS Expression in RAW 264.7 Cells

**Figure 1:**
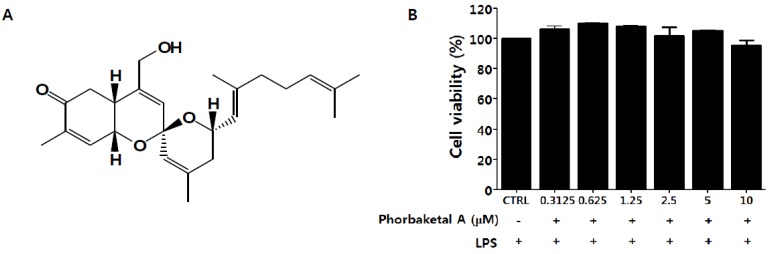

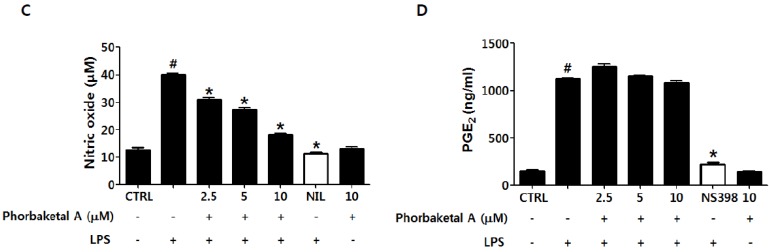
Chemical structures of phorbaketal A and its effects on production of nitric oxide (NO) and prostaglandins E_2_ (PGE_2_) in lipopolysaccharide (LPS)-stimulated RAW 264.7 cells. (**A**) Structures of phorbaketal A; (**B**–**D**) RAW 264.7 cells were pretreated with different concentrations of phorbaketal A (2.5, 5, and 10 μM) for 1 h and then stimulated with LPS (1 μg/mL) for 24 h; (**B**) Cell viability was determined by MTT assays; (**C**) The nitrite which accumulated in culture medium was measured as an indicator of NO production according to the Griess method. l-N6-(1-iminoehyl)lysine (l-NIL) (10 μM) was used as assay positive controls for NO production; (**D**) Levels of PGE_2_ were measured using the EIA kit. NS398 (10 μM) was used as assay positive controls for PGE_2_ production. Data are presented as the means ± SD of three independent experiments. Statistical analysis was carried out using the one-way ANOVA followed by the Tukey’s test. ^#^ *p* < 0.05 *vs.* CTRL group. * *p* < 0.05 *vs.* LPS-stimulated group.

**Figure 2:**
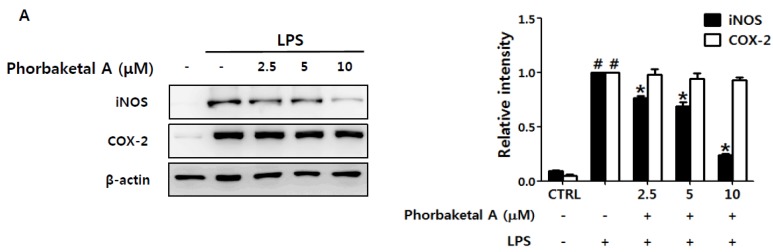

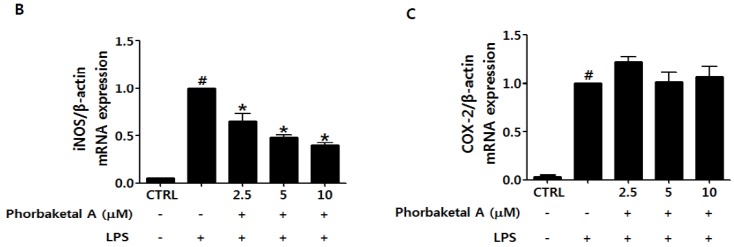
Effects of phorbaketal A on the expression of inducible nitric oxide synthase (iNOS) and cyclooxygenase-2 (COX-2) in LPS-stimulated RAW 264.7 cells. (**A**) RAW 264.7 cells were pretreated with different concentrations of phorbaketal A (2.5, 5, and 10 μM) for 1 h and then stimulated with LPS (1 μg/mL) for 24 h. Protein levels of iNOS, COX-2, and β-actin were determined by Western blot analysis. β-Actin was used as an internal control. The data shown are representative of three separate experiments; (**B**–**C**) RAW 264.7 cells were pretreated with different concentrations of phorbaketal A (2.5, 5, and 10 μM) for 1 h and then stimulated with LPS (1 μg/mL) for 8 h. The mRNA levels of iNOS (**B**) and COX-2 (**C**) were measured by real-time RT-PCR. Data are presented as the means ± SD of three independent experiments. Statistical analysis was carried out using the one-way ANOVA followed by the Tukey’s test. ^#^ *p* < 0.05 *vs.* CTRL group. * *p* < 0.05 *vs.* LPS-stimulated group. ^#^ *p* < 0.05 *vs.* CON group. * *p* < 0.05 *vs.* LPS-stimulated group.

We first evaluated the inhibitory effects of phorbaketal A (Figure 1A), isolated from the marine sponge *Phorbas* sp., on the production of two key inflammatory mediators, NO and PGE_2_, in macrophages. Based on MTT (3-(4,5-dimethylthiazol-2-yl)-2,5-diphenyltetrazolium bromide) assay data (Figure 1B), concentrations of phorbaketal A that would not affect cell viability (2.5, 5, and 10 μM) were used for following experiments. Phorbaketal A significantly and dose-dependently suppressed NO production in LPS-stimulated RAW 264.7 cells (Figure 1C). In contrast, treatment with phorbaketal A did not modify PGE_2_ production (Figure 1D). l-N6-(1-iminoehyl)lysine (10 μM) and NS398 (10 μM) were used as inhibitors of NO and PGE_2_ production, respectively. We next investigated whether the inhibitory effects of phorbaketal A on NO production were associated with regulation of the expression of inducible NO synthase (iNOS). Western blotting revealed that phorbaketal A significantly suppressed LPS-induced iNOS expression at the protein level (Figure 2A). In addition, real-time RT-PCR analysis revealed that phorbaketal A markedly decreased the mRNA expression of iNOS (Figure 2B). Notably, phorbaketal A did not induce a significant change in either the mRNA or protein level of cyclooxygenase-2 (COX-2), an enzyme involved in the synthesis of PGE_2_ (Figure 2A,C). These observations suggest that phorbaketal A significantly suppressed NO production by inhibiting iNOS expression at the transcriptional level, and that it had little effect on PGE_2_ and COX-2.

### 2.2. Phorbaketal A Inhibits the LPS-Induced Expression of Pro-Inflammatory Cytokines in RAW 264.7 Cells

**Figure 3:**
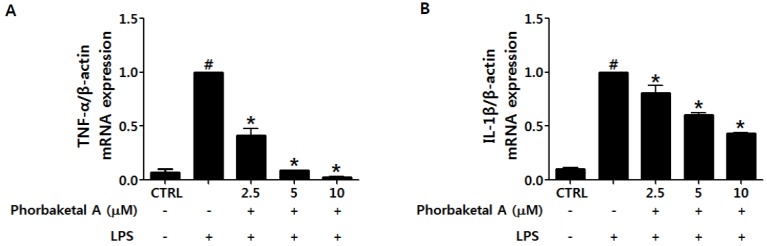

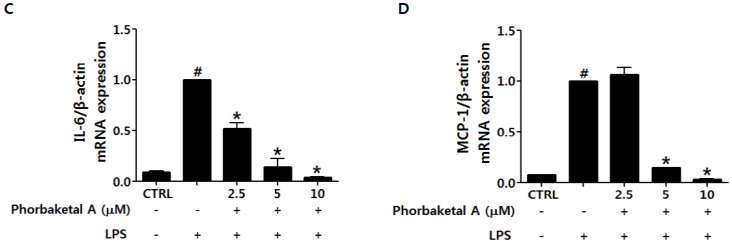
Effects of phorbaketal A on the expression of pro-inflammatory cytokines in LPS-stimulated RAW 264.7 cells. (**A**–**D**) RAW 264.7 cells were pretreated with different concentrations of phorbaketal A (2.5, 5, and 10 μM) for 1 h and then stimulated with LPS (1 μg/mL) for 8 h. The mRNA levels of tumor necrosis factor-alpha (TNF-α) (**A**); interleukin-1beta (IL-1β) (**B**); IL-6 (**C**); and monocyte chemotactic protein-1 (MCP-1) (**D**) were determined by real-time RT-PCR. Data are presented as the means ± SD of three independent experiments. Statistical analysis was carried out using the one-way ANOVA followed by the Tukey’s test. ^#^ *p* < 0.05 *vs.* CTRL group. * *p* < 0.05 *vs.* LPS-stimulated group.

Upon activation, macrophages promote inflammatory processes via the release of diverse pro-inflammatory cytokines, including TNF-α, IL-1β, IL-6, and MCP-1 [2,30]. TNF-α, IL-1β, and IL-6 are elevated in most inflammatory states and are known targets of anti-inflammatory therapeutic agents [31]. MCP-1 has been suggested to induce monocyte recruitment [32]. We investigated the effect of phorbaketal A on the LPS-induced expression of TNF-α, IL-1β, IL-6, and MCP-1 using real-time RT-PCR in RAW 264.7 cells. As shown in Figure 3, treatment with phorbaketal A markedly suppressed the expression of TNF-α, IL-1β, IL-6, and MCP-1 in a dose-dependent manner.

### 2.3. Phorbaketal A Inhibits the LPS-Induced Transcriptional Activity of NF-κB in RAW 264.7 Cells

**Figure 4:**
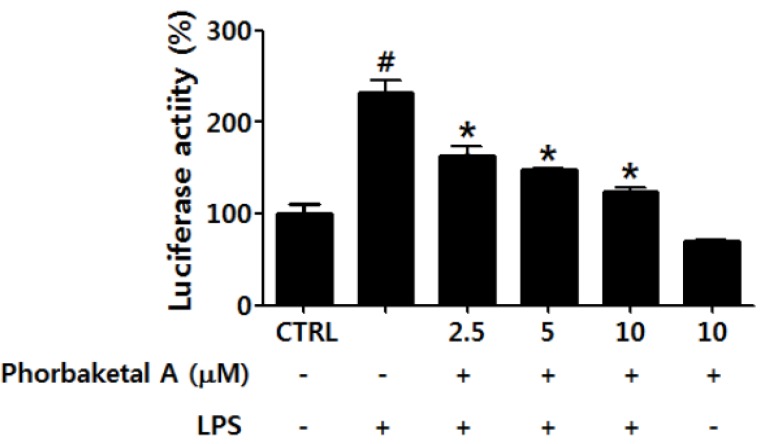
Effects of phorbaketal A on the activation of nuclear factor-kappaB (NF-κB) in LPS-stimulated RAW 264.7 cells. Cells were transiently transfected with pNF-κB-Luc vector, and then pretreated with different concentrations of phorbaketal A (2.5, 5, and 10 μM) for 1 h followed by LPS (1 μg/mL) stimulation for 2 h. Then luciferase activities were determined using a Promega luciferase assay system. Data are presented as the means ± SD of three independent experiments. Statistical analysis was carried out using the one-way ANOVA followed by the Tukey’s test. ^#^ *p* < 0.05 *vs.* CTRL group. * *p* < 0.05 *vs.* LPS-stimulated group.

NF-κB is a key transcription factor in immune and inflammatory responses. It plays a major role in inflammatory processes by regulating the expression of pro-inflammatory genes, including those encoding iNOS, TNF-α, IL-1β, and IL-6 [33,34,35]. When NF-κB is stimulated with inflammatory agents such as LPS, activated NF-κB dimers (of which p50/p65 is the most common heterodimer) translocate to the nucleus and interact with target DNA recognition sites to activate the transcription of diverse pro-inflammatory genes [36,37]. To test whether the anti-inflammatory effects of phorbaketal A are associated with the inhibition of NF-κB activation in RAW 264.7 cells, the transcriptional activity of NF-κB was evaluated using a luciferase assay. As shown in Figure 4, phorbaketal A significantly suppressed the LPS-induced transcriptional activity of NF-κB in LPS-stimulated macrophages. These results suggest that phorbaketal A inhibits the expression of pro-inflammatory genes, including those encoding iNOS, TNF-α, IL-1β, IL-6, and MCP-1, via negative regulation of the NF-κB pathway.

Previous studies have reported that oxidative stress leads to the activation of the NF-κB and this activation could be inhibited by antioxidants [38,39,40,41]. Lee *et al.* [42] demonstrated that antioxidants inhibit phosphorylation of IκBα and DNA-binding activity of NF-κB. In addition, it was reported that overexpression of an antioxidant enzyme superoxide dismutase (SOD), which can modulate intracellular ROS levels, plays an important role in NF-κB activation [43]. Similarly, Li, X.H. *et al.* [44] also demonstrated that DHCR24, the antioxidant enzyme, can block NF-κB activation by various stimuli. In this regard, it is possible that the anti-inflammatory effects of phorbaketal A on suppression of NF-κB activation could be mediated, at least partly, by suppressing oxidative stress. Further research should evaluate this possibility.

### 2.4. Phorbaketal A Regulates the HO-1 Pathway in RAW 264.7 Cells

**Figure 5:**
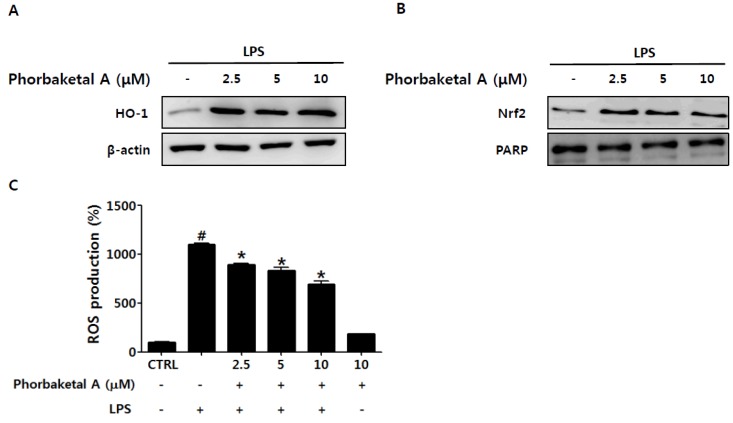
Effects of phorbaketal A on the nuclear transcription factor erythroid 2-related factor 2 (Nrf2)/heme oxyganase-1 (HO-1) pathway and ROS production in RAW 264.7 cells. (**A**) RAW 264.7 cells were pretreated with different concentrations of phorbaketal A (2.5, 5, and 10 μM) for 1 h and then stimulated with LPS (1 μg/mL) for 24 h. Protein levels of HO-1 and β-actin were determined in the whole protein lysate by Western blot analysis. β-Actin was used as an internal control. The data shown are representative of three separate experiments; (**B**) RAW 264.7 cells were pretreated with different concentrations of phorbaketal A (2.5, 5, and 10 μM) for 1 h and then stimulated with LPS (1 μg/mL) for 16 h. Western blot analysis was performed to detect Nrf2 in the nuclear fraction of the cell lysate. Nuclear protein PARP was used as an internal control. The data shown are representative of three separate experiments; (**C**) RAW 264.7 cells were pretreated with different concentrations of phorbaketal A (2.5, 5, and 10 μM) for 1 h and then stimulated with LPS (1 μg/mL) for 24 h. DCFH-DA (10 μM) was added and incubated for 30 min at 37 °C. DCF fluorescence by reactive oxygen species (ROS) was measured by flow cytometry. Data are presented as the means ± SD of three independent experiments. Statistical analysis was carried out using the one-way ANOVA followed by the Tukey’s test. ^#^ *p* < 0.05 *vs.* CTRL group. * *p* < 0.05 *vs.* LPS-stimulated group.

In addition to inhibition of the NF-κB pathway, up-regulation of the Nrf2/HO-1 pathway has been suggested as a key mechanism of various anti-inflammatory activities [7,8]. HO-1 and its products exert anti-inflammatory effects via attenuation of the expression of pro-inflammatory mediators such as NO, PGE_2_, TNFα, IL-1β, IL-6, and MCP-1 in activated macrophages [10,11,12,13]. In addition, HO-1 has been demonstrated to inhibit pro-inflammatory responses in activated macrophages through modulation of NF-κB activation [11,13,45]. Thus, we next examined whether phorbaketal A induces HO-1 expression in LPS-stimulated RAW 264.7 cells. Phorbaketal A markedly increased HO-1 protein expression in RAW 264.7 cells (Figure 5A). Considering that HO-1 is induced by the transcription factor Nrf2 [46], we investigated whether phorbaketal A is able to induce the nuclear translocation of Nrf2. Phorbaketal A increased the nuclear translocation of Nrf2 (Figure 5B), suggesting that the induction of HO-1 by phorbaketal A is associated with Nrf2 activation. It is well known that the Nrf2/HO-1 pathway involves ROS generation. ROS have been proposed to be key signaling molecules in LPS-induced inflammation [47,48]. In fact, during inflammation, activated macrophages show massive ROS accumulation. ROS can stimulate cellular activities such as cytokine secretion and cell death [49]. Therefore, we examined the effect of phorbaketal A on LPS-induced ROS accumulation in RAW 264.7 cells. As shown in Figure 5C, phorbaketal A significantly reduced ROS accumulation in a dose-dependent manner. Thus, the anti-inflammatory effects of phorbaketal may be associated with its anti-oxidant property. Additional studies are needed to reveal the definitive molecular mechanisms.

## 3. Experimental Section

### 3.1. Preparation of Phorbaketal A

Phorbaketal A (Figure 1A) (>96% purity) used for this study was isolated from the marine sponge *Phorbas* sp. off the shore of Gageo Island, South Korea, [27]. As previously described [27], the sponge was extracted twice with MeOH and then partitioned between CH_2_Cl_2_ and H_2_O. The organic layer was further repartitioned between 15% aqueous MeOH and *n*-hexane. After fractionation of the polar layer and continuous HPLC processes, phorbaketal A was purified as a yellowish oil. Phorbaketal A was identified as sesterterpenoid with a spiroketal of hydrobenzopyran moiety by spectral and chemical method.

### 3.2. Materials

Dulbecco’s modified Eagle’s minimum essential medium (DMEM), fetal bovine serum (FBS), penicillin, and streptomycin were obtained from Life Technologies Inc. (Grand Island, NY, USA). COX-2, iNOS, HO-1, Nrf2, β-actin, and PARP antibodies were purchased from Santa Cruz Biotechnology, Inc. (Santa Cruz, CA, USA). The enzyme immunoassay (EIA) kits for PGE_2_ were obtained from R&D Systems (Minneapolis, MN, USA). The RNA extraction kit was purchased from Intron Biotechnology (Seoul, Korea). The luciferase assay kit was purchased from Promega (Madison, CA, USA). The p-NF-κB-Luc reporter plasmid was purchased from BD Biosciences (San Diego, CA, USA). The COX-2, iNOS, TNF-α, IL-1β, IL-6, MCP-1, and β-actin oligonucleotide primers were purchased from Bioneer (Seoul, Korea). 3-(4,5-Dimethylthiazol-2-yl)-2,5-diphenyl-tetrazo-liumbromide (MTT), phenyl methyl sulfonylfluoride (PMSF), l-N6-(1-iminoethyl)lysine (l-NIL), methanesulfonamide (NS398), LPS (*Escherichia coli*, serotype 0111:B4), and all other chemicals were purchased from Sigma Chemical Co.(St. Louis, MO, USA).

### 3.3. Cell Culture and Sample

RAW264.7 macrophages were obtained from the Korea Cell Line Bank (Seoul, Korea). Cells were grown at 37 °C in DMEM medium supplemented with 10% FBS, penicillin sulfates in a humidified atmosphere of 5% CO_2_. Cells were pretreated with phorbaketal A (2.5, 5, or 10 μM) or positive controls (l-NIL for iNOS inhibitor or NS398 for COX-2 inhibitor) for 1 h, and then stimulated with LPS (1 μg/mL) for the indicated time.

### 3.4. Cell Viability Assay

Cell viability studies were performed using the MTT (3-[4,5-dimethylthiazol-2yl]-2,5-dipheyl tetrazoliumbromide; Sigma-Aldrich) assay. Raw 264.7 cells were plated at a density of 1 × 10^5^ cells/mL in 96-well. Cells were pretreated with phorbaketal A (2.5, 5, or 10 μM) for 1 h and then stimulated with LPS (1 μg/mL) for 24 h. 50 μL of MTT solution (5 mg/mL in PBS) was added to the medium and the cells were incubated at 37 °C for 4 h. The MTT-containing medium was removed and the cells were solubilized in DMSO (100 μL) for 10 min. The optical density at 540 nm was determined using a microplate spectrophotometer (Molecular Devices Inc., Sunnyvale, CA, USA) to determine the cell viability.

### 3.5. Determination of NO Production

The nitrite which accumulated in culture medium was measured as an indicator of NO production according to the Griess method. Briefly, 100 μL of cell culture medium was mixed with 100 μL of Griess regent (equal volumes of 1% (*w*/*v*) sulfanilamide in 5% (*v*/*v*) phosphoric acid and 0.1% (*w*/*v*) naphtylethylenediamine-HCl), incubated at room temperature for 5 min, and then the absorbance at 540 nm was determined in a microplate reader (Molecular Devices Inc., Sunnyvale, CA, USA). The amount of nitrite in the samples was determined with reference to a sodium nitrite standard curve.

### 3.6. Determination of PGE_2_ Production

Levels of PGE_2_ in the culture media were determined using EIA kits according to the manufacturer’s instructions (R&D Systems).

### 3.7. Real-Time RT-PCR Analysis

Total cellular RNA was isolated using Easy Blues kits (Intron Biotechnology) according to the manufacturer’s instructions. For each sample, 1 mg of RNA was reverse-transcribed (RT) using MLV reverse transcriptase, 1 mM dNTP, and oligo (dT12–18) 0.5 mg/mL. Real-time RT-PCR was performed using Thermal Cycler Dice Real Time PCR System (Takara, Japan). The primers used for SYBR Green real-time RT-PCR were as follows: for iNOS, sense primer, 5′-CAT GCT ACT GGA GGT GGG TG-3′ and anti-sense primer, 5′-CAT TGA TCT CCG TGA CAG CC-3′; for COX-2, sense primer, 5′-TGC TGT ACA AGC AGT GGC AA-3′ and anti-sense primer, 5′-GCA GCC ATT TCC TTC TCT CC-3′; for TNF-α, sense primer, AGC ACA GAA AGC ATG ATC CG-3′ and anti-sense primer, 5′-CTG ATG AGA GGG AGG CCA TT-3′; for IL-1β, sense primer, 5′-ACC TGC TGG TGT GTG ACG TT-3′ and anti-sense primer, 5′-TCG TTG CTT GGT TCT CCT TG-3′; for IL-6, sense primer, 5′-GAG GAT ACC ACT CCC AAC AGA CC-3′ and anti-sense primer, 5′-AAG TGC ATC ATC GTT GTT CAT ACA-3′; for MCP-1, sense primer, 5′-GCA TCC ACG TGT TGG CTC A-3′ and anti-sense primer, 5′-CTC CAG CCT ACT CAT TGG GAT CA-3′; for β-actin, sense primer, 5′-ATC ACT ATT GGC AAC GAG CG-3′ and anti-sense primer, 5′-TCA GCA ATG CCT GGG TAC AT-3′. PCRs were carried out for 50 cycles using the following conditions: denaturation at 95 °C for 5 s, annealing at 57 °C for 10 s, and elongation at 72 °C for 20 s. The mean Ct of the gene of interest was calculated from triplicate measurements and normalized with the mean Ct of a control gene, β-actin.

### 3.8. Preparation of Nuclear Fractions

RAW264.7 cells were plated in 60 mm dishes (2 × 10^5^ cells/mL), pretreated with phorbaketal A for 1 h and then stimulated with LPS (1 μg/mL) for 16 h. The cells were washed once with phosphate-buffered saline (PBS), scraped into 1 mL of cold PBS, and pelleted by centrifugation. The cell pellets were resuspended in hypotonic buffer (10 mM HEPES, pH 7.9, 1.5 mM MgCl_2_, 10 mM KCl, 0.2 mM PMSF, 0.5 mM DTT, 10 μg/mL aprotinin) and incubated on ice for 15 min. The cells were then lysed by adding 0.1% Nonidet P-40 and vortexed vigorously for 10 s. Nuclei were pelleted by centrifugation at 12,000× *g* for 1 min at 4 °C and resuspended in high salt buffer (20 mM HEPES, pH 7.9, 25% glycerol, 400 mM KCl, 1.5 mM MgCl_2_, 0.2 mM EDTA, 0.5 mM DTT, 1 mM NaF, 1 mM sodium orthovanadate).

### 3.9. Western Blot Analysis

Cells were collected by centrifugation and washed once with PBS. The washed cell pellets were re-suspended in an extraction lysis buffer (50 mM HEPES pH 7.0, 250 mM NaCl, 5 mM EDTA, 0.1% Nonidet P-40, 1 mM phenylmethylsulfonyl fluoride, 0.5 mM dithiothreitol, 5 mM sodium fluoride and 0.5 mM sodium orthovanadate) containing 5 μg/mL of leupeptin and 5 μg/mL of aprotinin and incubated for 20 min at 4 °C. Cell debris was removed by micro-centrifugation. The protein concentration was determined using the Bio-Rad protein assay reagent according to the manufacture’s instruction. Cellular protein (15 μg) was electroblotted onto a PVDF membrane following separation on a SDS-polyacrylamide gel electrophoresis. The membranes were blocked in 5% skim milk for 30 min, and then incubated with a 1:1000 dilution of specific antibodies against iNOS, COX-2, HO-1, NRf2, and β-actin in Tris-buffered saline (TBS) containing Tween-20 (0.1%) overnight at 4 °C. Primary antibodies were removed by washing membranes three times in TBS-T, and then the membranes were incubated with a 1:1000 dilution of horseradish peroxidase-conjugated secondary antibody for 2 h at room temperature. Following three washes in TBS-T, immunopositive bands were visualized by enhanced chemiluminescence (Amersham Life Science, Arlington Heights, IL, USA) and exposed to ImageQuant LAS-4000 (Fujifilm Life Sceince, Tokyo, Japan).

### 3.10. Transfection and Luciferase Assay

RAW264.7 cells were co-transfected with mouse NF-κB-luc reporter plasmid and phRL-TK plasmid (Promega, Madison, WI, USA) using Lipofectamine LTX™ (Invitrogen, Carlsbad, CA, USA) according to the manufacturer’s instruction. At 24 h after transfection, cells were pretreated with phorbaketal A for 1 h and then stimulated with LPS (1 μg/mL) for 2 h. The cells were lysed and the luciferase activities were determined using the Promega luciferase assay system (Promega, Madison, WI, USA) according to the manufacturer’s instructions.

### 3.11. Detection of ROS Production

RAW264.7 cells were pretreated with phorbaketal A for 1 h, and then stimulated with LPS (1 μg/mL) for 24 h. After 24 h of stimulation, DCFH-DA (10 μM) was incubated for 30 min. The medium was removed and the cells were washed 3 times with PBS. The capacity of cells to produce ROS was measured by DCF fluorescence using flow cytometry.

### 3.12. Statistical Analysis

Statistical data are presented as the mean ± SD of three individual experiments preformed in triplicate. Statistical analysis was carried out using the one-way ANOVA followed by the Tukey’s test. ^#^ *p* < 0.05 *vs.* CTRL group. * *p* < 0.05 *vs.* LPS-stimulated group.

## 4. Conclusions

Marine natural products have great biological and chemical diversity. Marine sponges of the genus *Phorbas* are known to produce diverse and potent biologically active components with unique structures [26]. Phorbaketal A is a tricyclic sesterterpenoid isolated from the marine sponge *Phorbas* sp. Sesterterpenoids are isoprenoid natural products that have been isolated from a range of sources, including terrestrial fungi, higher plants, lichens, insects, and marine invertebrates [50,51]. Sponges are the richest source of marine sesterterpenoids [52]. A wide variety of pharmaceutically relevant biological activities, including antibiotic, anticancer, and anti-inflammatory activities, have been reported for sesterterpenoids [50,51,53,54,55]. However, few studies evaluated the biological activities of the sesterterpenoid phorbaketal A, isolated from the marine sponge *Phorbas* sp., and thus its molecular mechanism remains poorly understood.

In this study, we demonstrated that phorbaketal A inhibits the release of NO and the expression of iNOS and pro-inflammatory cytokines in LPS-stimulated macrophage. The anti-inflammatory properties of phorbaketal A seem to be mediated by the HO-1 induction and down-regulation of the NF-κB pathway. To the best of our knowledge, this is the first comprehensive study on anti-inflammatory effect of phorbaketal A and its molecular mechanism of action in macrophage. Overall, our data suggest a potential therapeutic value of phorbaketal A to be further developed as novel anti-inflammatory drug.
